# Four-year prospective evaluation of the relationship between meaning in life and smoking status

**DOI:** 10.1186/1747-597X-8-8

**Published:** 2013-02-22

**Authors:** Barna Konkolÿ Thege, Róbert Urbán, Mária S Kopp

**Affiliations:** 1 Institute of Behavioral Sciences, Semmelweis University, Nagyvárad tér 4. XX. em, Budapest, Hungary; 2 Department of Psychology, University of Calgary, 2500 University Drive NW, Calgary, AB T2N1N4, Canada; 3 Department of Personality and Health Psychology, Eötvös Loránd University, Izabella u. 46, Budapest, 1078, Hungary

**Keywords:** Change in smoking status, Meaning in life, Prospective design, Structural equation modeling

## Abstract

**Background:**

To date, all investigations on the relationship between smoking and perceived level of meaning in life have used cross-sectional designs. Therefore, the purpose of the present prospective study, conducted with a four-year time lag, was to test the predictive power of the life meaning construct concerning changes in smoking status.

**Methods:**

The data of 4,294 respondents (40.3% male, M_age_ = 54.7 ± 16.5 yrs) from the Hungarian Epidemiological Panel Survey were analyzed using the Kruskal-Wallis and Mann–Whitney *U*-test and structural equation modeling (SEM) with a nominal outcome variable. Gender, age, and educational level were included in the study as covariates.

**Results:**

On the bivariate level, results showed that both baseline and follow-up meaning in life scores were higher in stable non-smokers when compared to stable smokers. However, quitters and starters differed from stable non-smokers in their baseline but not in follow-up life meaning scores. The other relationships (stable smokers vs. quitters; stable smokers vs. starters, starters vs. quitters) were non-significant in both time points. According to the SEM-analysis, a higher sense of meaning in life measured at baseline and follow-up is associated with a lower likelihood (OR = 0.54, z = 2.80, p = 0.005; OR = 0.64, z = 2.88, p = 0.004, respectively) of being a stable smoker compared to being a stable non-smoker, confirming the expected relationship between smoking and decreased level of meaning in life. However, neither baseline nor follow-up life meaning scores predicted significantly quitting and uptake of smoking.

**Conclusions:**

If future research from other cultures verifies the protective role of a higher level of meaning in life against smoking, then smoking prevention and cessation programs will also have to include such components that help individuals experience more meaning in their lives.

## Background

The self-medication theory of smoking emphasizes the role of coping with negative emotions and unpleasant psychological states as anxiety, boredom and distress in cigarette use [1]. According to some recent studies, the decreased level of perceived meaning in life is also a negative state that can facilitate smoking [2-5]. Issues considered integral to life meaning are having a sense of clear aims, a sense of achieving life goals, and a feeling that one’s experiences and daily activities are worthwhile and meaningful [6]. Personal meaning is the complex of connections, understandings, and interpretations that help the person comprehend his/her experience and formulate plans directing energies to the achievement of the desired future [7]. According to Frankl [8], people are capable of discovering meaning in their lives through the actualization of different values that can be creative (occupation, any kind of active deeds that create something valuable), experiential (appreciation of anything that the person values: art, nature, humor, love of others) or attitudinal (e.g. facing uncontrollable factors such as illness or death with dignity).

Having a weaker sense of purpose and meaning in life results in an increased proneness to boredom [9] and an enlarged sensitivity to societal pressures [8], thereby raising the probability of cigarette use [10], especially in those societies where attitudes toward smoking are permissive or encouraging. According to the previously cited studies, smokers also seem to perceive their lives as less meaningful compared to non-smokers—similarly to the users of other legal [6] or illegal substances [11]. However, all of the examinations on this topic used cross-sectional designs; not allowing to test the predictive power of the life meaning construct regarding smoking behavior. The aim of the present study, therefore, was to examine whether meaning in life predicts smoking status in a longitudinal analysis.

## Methods

### Sample and procedure

The Hungarian Epidemiological Panel (HEP) is a prospective survey focused on the quality of life and the biopsychosocial causes of the development and progress of diseases of public health importance in the Hungarian adult population. After receiving the approval of the Ethical Committee of Semmelweis University, the first wave of the data collection was conducted in 2002 where the sampling frame was the National Population Register. Towns with a population of more than 10,000, as well as a random sample of smaller villages were included in the sample.

At follow-up, approximately 4 years later, 4,524 adult (22 years of age or older) persons were interviewed face-to-face in the survey, when the sampling frame was limited to those who gave consent in 2002 to participate in the second phase. The refusal rate was 23.7%. Those refusing to participate in the follow-up were more likely males (*χ*^2^ = 18.17; p < 0.001), older (U = 3635905.0; p < 0.001), and more educated (U = 3378401.0; p < 0.001); however, they did not differ significantly from respondents of the second data wave concerning smoking status (*χ*^2^ = 1.16; p = 0.282) and perceived level of meaning in life (U = 3745265.5; p = 0.386). These data suggest that attrition might not have affected substantially the generalizability of our findings.

The representativity of the sample by age, gender, and sub-region was obtained by a weighting process. The sampling methods are described in detail elsewhere [12]. In case of missing data or inconsistent responses (those who reported on being a former or current smoker at baseline and a never smoker at follow-up), participants were eliminated from the analyses (4.8%); therefore, the final sample size of this study was 4,294 (40.3% male, M_age-at-baseline_ = 54.7 ± 16.5 yrs).

### Measures

The following variables of the complex test battery from the HEP Survey were used in the present study: participants’ gender, age, educational level (six answer categories from less than primary to university level), smoking status (current smoker vs. current non-smoker), and perceived level of meaning in life. Smoking status was originally assessed by the question: ‘Do you smoke cigarettes?’^a^ with three answer categories: No, never; Currently no, but previously I did; Yes, I do. Perceived meaning in life was measured by the seven-item Hungarian version of the Life Meaning Subscale [13] from the Brief Stress and Coping Inventory [14]. This questionnaire includes items such as “I feel my life is part of a larger plan”, “My values and beliefs guide me daily” or “I doubt that my life makes a difference” (reverse coded). The scale has a three-point rating scale: 0 – rarely, 1 – sometimes, 2 – often. Internal consistency of the scale was acceptable in this sample (α_T1_ = .67; α_T2_ = .75).

### Statistical analyses

Considering smoking status, we created a nominal variable coding four groups, namely (1) stable smokers who smoked both at T1 and T2; (2) quitters who smoked at T1 and did not smoke at T2; (3) starters who initiated smoking by T2 but did not smoke at T1; (4) stable non-smokers who smoked neither at T1 nor T2. We did not distinguish those who started to smoke by T2 from those relapsed back to smoking for statistical power reasons.

At the bivariate level, the SPSS 20.0 software was employed and after the Kruskal-Wallis test, the Mann–Whitney test was used to analyze the relationship between meaning in life and change in smoking status. Strength of the relationships was expressed by calculating r coefficients (r = Z/√N).

For the multivariate analyses, we applied the structural equation modeling framework [15,16]. Temporal stability of our life meaning measure was tested with a multiindicator autoregressive model estimated with MPLUS 6.0 software [17]. The multivariate model tested the simultaneous effects of baseline and follow-up meaning in life (as latent variables), gender, age, and educational level on smoking status change. This time, we also employed MPLUS 6.0 using maximum likelihood parameter estimates with standard errors and chi-square test statistics that are robust to non-normality.

## Results

As a preliminary examination, we performed two separate SEM analyses for T1 and T2 to confirm previous findings on the cross-sectional associations between smoking status (current smoker vs. current non-smoker) and life meaning. In these models, life meaning was used as a latent continuous independent variable, while smoking status as an observed dichotomous dependent variable. Life meaning was significantly associated with smoking status at both T1 (OR = 0.51, CI = 0.42-0.65, z = 3.48, p < 0.001) and T2 (OR = 0.61, CI = 0.52-0.70, z = 3.60, p < 0.001) providing further evidence that a higher level of perceived meaning in life is associated with a lower probability of smoking.

The examination of the proportion of the groups according to smoking status change revealed that 66.2% of the participants were stable non-smokers, 23.7% were stable smokers, 4.7% were quitters, while 5.4% were starters. When testing the temporal stability of our meaning in life measure, the fit indices [*χ*^2^ = 593.9, df = 69, CFI = 0.97, TLI = 0.96, RMSEA = 0.042 (0.039-0.045)] indicated an excellent level of fit of our model providing evidence for the stability of the measurement model in the two measurement occasions. The correlation coefficient between meaning in life scores at T1 and T2 was 0.27 (p < 0.001).

Bivariate level associations between meaning in life and smoking status change are displayed in Table 1. First, the Kruskal-Wallis test was used to determine, whether any relationship existed between meaning in life and smoking status change. Since the results showed that the two constructs were related, pairwise comparisons were conducted using the Mann–Whitney test. These analyses revealed that both baseline and follow-up meaning in life scores were significantly higher in stable non-smokers when compared to stable smokers. However, quitters and starters differed from stable non-smokers in their life meaning scores only at T1 but not at T2. In all cases, the associations proved to be weak. The other relationships (stable smokers vs. quitters; stable smokers vs. starters, starters vs. quitters) were non-significant in both time points.

**Table 1:** Baseline and follow-up meaning in life scores [M(SD)] stratified by smoking status

		**Baseline meaning in life scores**	**Follow-up meaning in life scores**
Stable non-smokers (a)		10.21 (2.57)	10.02 (2.73)
Stable smokers (b)		9.66 (2.70)	9.54 (2.92)
Starters (c)		9.81 (2.62)	9.78 (2.85)
Quitters (d)		9.85 (2.66)	9.74 (3.05)
Kruskall-Wallis one-way analysis of ranks		*χ*^2^ = 35.11	*χ*^2^ = 19.61
		df = 3	df = 3
		p < 0.001	p < 0.001
Multiple pairwise comparisons with Mann–Whitney test	a vs. b	U = 1236219.5; p < 0.001; r = 0.09	U = 1306015.0; p < 0.001; r = 0.07
	a vs. c	U = 286889.0; p = 0.028; r = 0.04	U = 312517.5; p = 0.279; r = 0.02
	a vs. d	U = 254983.5; p = 0.049; r = 0.04	U = 271433.0; p = 0.291; r = 0.02
	b vs. c	U = 108765.0; p = 0.382; r = 0.02	U = 111752.0; p = 0.237; r = 0.03
	b vs. d	U = 96185.0; p = 0.405; r = 0.02	U = 97662.0; p = 0.306; r = 0.03
	c vs. d	U = 22362.5; p = 0.984; r < 0.01	U = 23121.0; p = 0.942; r < 0.01

At the multivariate level, a structural regression model was employed to examine the determinants of smoking status change. Due to our model specification, including an observed nominal outcome variable, the usual fit indices were not available to estimate the model fit. For that reason, the fit of our data can be expressed by loglikelihood estimates (−55115.529) and fit indices based on information criteria (Akaike information criterion = 110367.1, Bayesian information criterion = 110800.3, sample-size adjusted Bayesian information criterion = 110584.3). The predictors of smoking status change are reported in Table 2.

**Table 2:** Predictors of smoking status – odds ratios according to the structural regression model

	**Stable smokers**^**+ **^**(N = 1019)**	**Quitters**^**+ **^**(N = 201)**	**Starters**^**+ **^**(N = 231)**
Life meaning at T1	0.54^**^ [0.35-0.84]	0.56 [0.25-1.28]	0.55 [0.25-1.23]
Life meaning at T2	0.64^**^ [0.47-0.87]	0.63 [0.35-1.16]	0.71 [0.40-1.25]
Gender (1 – ♂; 2 – ♀)	0.52^***^ [0.44-0.61]	0.50^***^ [0.37-0.68]	0.67^**^ [0.51-0.89]
Age	1.04^***^ [1.03-1.05]	1.03^***^ [1.02-1.04]	1.06^***^ [1.05-1.07]
Level of education	0.80^***^ [0.75-0.85]	0.99 [0.89-1.11]	0.90 [0.81-1.01]

^+^Reference group: stable non-smokers (N = 2843); ^*^p < 0.05, ^**^p < 0.01, ^***^p < 0.001. Significance tests were carried out using Z-statistics (=coefficient estimate/standard error).

Our results have revealed that both baseline (OR = 0.54, CI = 0.35-0.84, z = 2.80, p = 0.005) and follow-up (OR = 0.64, CI = 0.47-0.87, z = 2.88, p = 0.004) meaning in life scores differentiated significantly between the stable non-smoker and the stable smoker groups again confirming that a stronger sense of meaning in life is associated with lower likelihood of smoking. Contrary to the bivariate analysis, these relationships were moderately strong. However, neither at T1 nor at T2 did our main independent variable predict significantly quitting or taking up smoking.

Concerning the other independent variables, the results showed that being male and older were associated with a higher likelihood of either current (stable smokers and starters) or previous (quitters) smoking. Further, higher level of education was a negative predictor only for stable smoking but not for being a quitter or starter.

## Discussion

The purpose of this study was to shed light on the predictive significance of meaning in life concerning smoking status. Consistent with our assumptions, having a stronger sense of meaning in life is related with a lower likelihood of stable smoking when compared to stable non-smoking. According to this part of the results, it seems reasonable to expand smoking prevention and cessation programs [18] with components to help individuals have a stronger sense of meaning in life [19,20].

Contrary to our expectations, however, perceived level of meaning in life did not distinguish between stable non-smokers, quitters, and starters. On one hand, this finding may be interpreted that the emergence or the lessening of existential concerns does not play a substantial role in the motivational background of initiating or giving up cigarette smoking. On the other hand, the number of quitters and starters was substantially lower in this study compared to stable smokers or non-smokers, which might also be a reason for the non-significant results concerning those who started or quitted smoking during the time lag of the study. A further possible explanation for the unexpected results is that the 4-year time-lag used was too long when considering the theoretically allowed [21] and empirically found [22] low stability of the meaning in life construct and the well-known fluctuation of smoking status (cf. cessation attempts and relapses through the smoking cessation process). According to this recognition, a further longitudinal study with more measurement points could more reliably follow the patterns of the associations between changes in life meaning and smoking status.

An additional important factor that may have considerably influenced the results is the age of respondents. In this study, the mean age of the respondents was approximately 55 years (with a standard deviation of about 17 years); that is, most of our respondents were middle-aged in which age group smoking initiation is uncommon in contrast with adolescence and young adulthood. It is likely that our results would follow a substantially different pattern, had this latter age group been investigated. To demonstrate this possibility, we can refer to the findings of Brassai and his colleagues where the data revealed that in adolescence, the *presence* of meaning in life did not [23] but the *search* for it did [24] act as a protective factor against smoking. These results should drive our attention to the importance of the consideration of different life stages when analyzing the relationships of existential constructs with health-related behaviors.

The findings presented in this paper must be interpreted in light of several limitations. Although the internal consistency of our life meaning measure was found to be quite good in several previous investigations [2,13], in the present study, the alpha value at baseline corresponded only to the acceptable range. However, this shortcoming affects mainly the analyses using the Kruskal-Wallis and the Mann–Whitney *U*-test, since throughout the other analyses, the meaning in life construct was used as a latent and not as an observed variable thus substantially decreasing measurement error (cf. the difference in the strength of the relationships revealed by the SEM and the more basic analyses). A further limitation of the reliability of our study is that we did not give a formal and detailed definition on smoking status categories to the participants and did not verify the data by biological indicators, either. Although this procedure is common and accepted when conducting large scale epidemiological studies as the HEP Survey reported here, it does not give very strict and unquestionable data on smoking behavior (cf. the relatively high rate of inconsistent responses).

It is also important to note that even if some of the samples previously used were large and nationally representative, all studies investigating the relationship between meaning in life and smoking—including the present one as well—were conducted in some Hungarian-speaking areas of Europe, namely in Hungary and Transylvania. Further examinations from other cultures should investigate the cultural independence of our findings.

## Conclusions

If future research from other cultures, aiming to better understand the role of existential concerns in nicotine use, verifies the protective role of a higher level of meaning in life against smoking, then smoking prevention and cessation programs will also have to include such components that help individuals find satisfying life purposes and meaning in their everyday experiences.

## Endnote

^a^Other tobacco products than cigarettes were not available in Hungary at the time of data collection.

## Competing interests

The authors declare that they have no competing interests.

## Authors’ contributions

BKT designed the study and managed the literature searches and analyses. BKT and RU conducted the statistical analysis. MK designed and managed the HEP 2006 research. BKT wrote the first draft of the manuscript. Both living authors read and approved the final manuscript.
